# The Impact of Dense Breasts on the Stage of Breast Cancer at Diagnosis: A Review and Options for Supplemental Screening

**DOI:** 10.3390/curroncol29050291

**Published:** 2022-05-17

**Authors:** Paula B. Gordon

**Affiliations:** Department of Radiology, Faculty of Medicine, University of British Columbia, 505-750 West Broadway, Vancouver, BC V5Z 1H4, Canada; pbgordon@mac.com

**Keywords:** mammography, breast ultrasound, breast density, breast cancer screening, interval cancer, mortality reduction, breast MRI, digital breast tomosynthesis, molecular breast imaging, contrast-enhanced mammography

## Abstract

The purpose of breast cancer screening is to find cancers early to reduce mortality and to allow successful treatment with less aggressive therapy. Mammography is the gold standard for breast cancer screening. Its efficacy in reducing mortality from breast cancer was proven in randomized controlled trials (RCTs) conducted from the early 1960s to the mid 1990s. Panels that recommend breast cancer screening guidelines have traditionally relied on the old RCTs, which did not include considerations of breast density, race/ethnicity, current hormone therapy, and other risk factors. Women do not all benefit equally from mammography. Mortality reduction is significantly lower in women with dense breasts because normal dense tissue can mask cancers on mammograms. Moreover, women with dense breasts are known to be at increased risk. To provide equity, breast cancer screening guidelines should be created with the goal of maximizing mortality reduction and allowing less aggressive therapy, which may include decreasing the interval between screening mammograms and recommending consideration of supplemental screening for women with dense breasts. This review will address the issue of dense breasts and the impact on the stage of breast cancer at the time of diagnosis, and discuss options for supplemental screening.

## 1. Background: Screening Mammography

The purpose of breast cancer screening is to find cancers early to reduce mortality and to allow successful treatment with less aggressive therapy. Mammography is the gold standard for breast screening. The randomized controlled trials (RCTs), carried out from the early 1960s to the mid 1990s showed a significant decrease in breast cancer deaths in women invited to screening. The magnitude of mortality reduction was shown to be considerably higher among women who actually participated in mammography screening regularly. Coldman et al. showed 40% mortality reduction overall, and 44% reduction in women aged 40–49 [1]. In Sweden, Tabár et al. showed that women aged 40–69 who participated in organized screening were 60% less likely to die of breast cancer in the 10 years following diagnosis, and 47% less likely within 20 years [2]. In a review of European studies, Broeders et al. showed that the relative reduction in breast cancer mortality for women who actually participated in screening was 38% based on incidence-based mortality studies and 48% based on case control studies [3].

## 2. What Is Breast Density?

**Figure 1:**
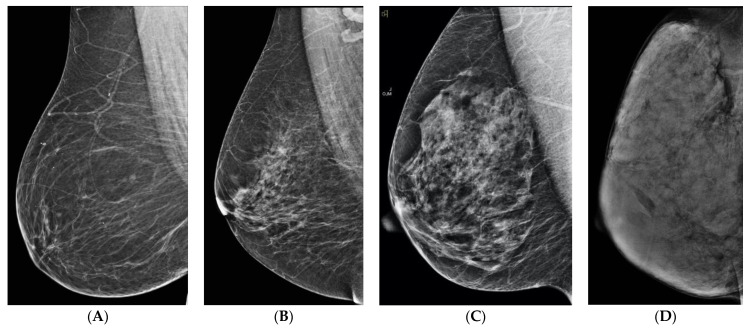
ACR BI-RADS 5th edition density categories: (**A**) Category A—almost entirely fat, (**B**) Category B—scattered fibroglandular densities, (**C**) Category C—heterogeneously dense, (**D**) Category D—extremely dense.

Breast density refers to the proportions of fibroglandular tissue and fat in women’s breasts, as seen on a mammogram. Unlike imaging of other organs, there is marked variability of the appearance of normal breast tissue, ranging from no dense tissue and almost all fat, to almost no fat, and all dense tissue. Breast density is divided into four categories in the American College of Radiology Breast Imaging and Data System (ACR BI-RADS) 5th edition. A—almost entirely fat, B—scattered fibroglandular densities, C—heterogeneously dense, D—extremely dense (Figure 1). Categories A and B are considered non-dense, and categories C and D are considered dense. Having dense breasts is normal and common: Among women aged 40 years or older, approximately 43% have dense breasts: 36% have Category C density, and 7% have Category D [4]. Han et al. found that women with a family history of breast cancer were more likely to have dense breasts than women with no family history [5].

## 3. What Is the Significance of Dense Breasts?

**Figure 2:**
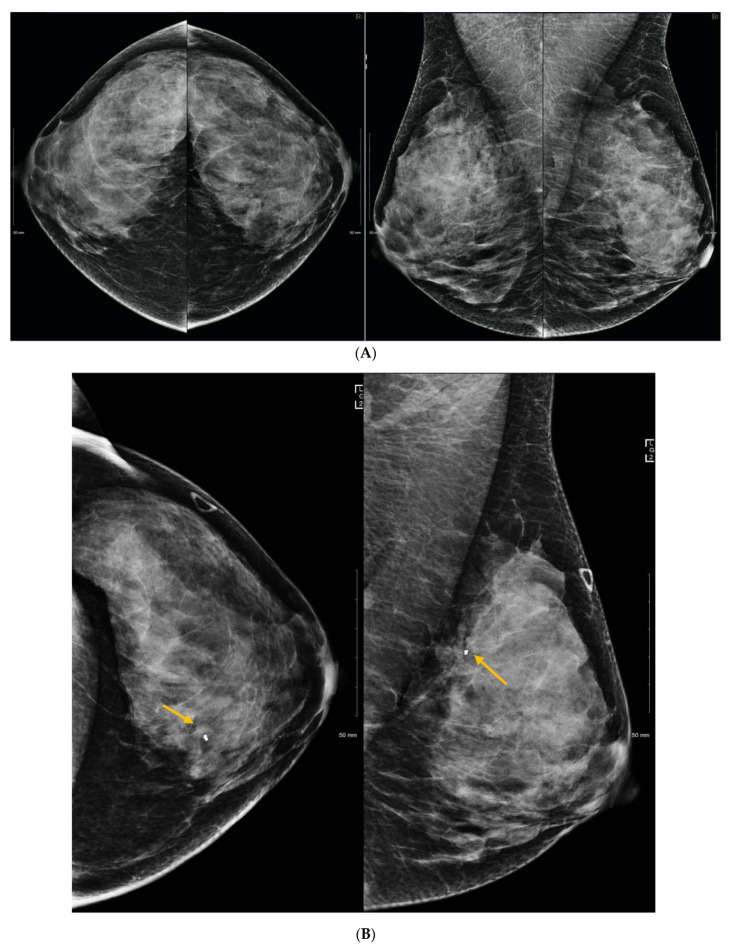

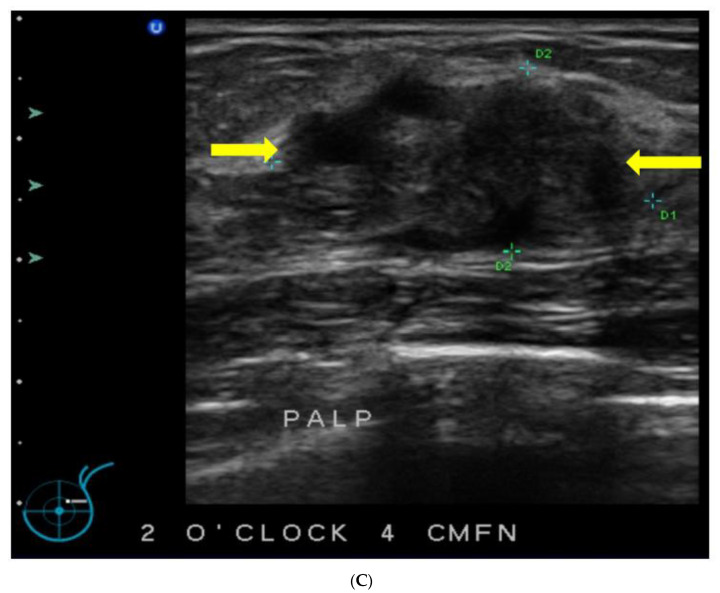
(**A**) Routine screening mammogram in a 50-year-old woman with screening Density Category D. Calcifications were seen in the left upper inner quadrant (not shown) for which stereotactic core biopsy showed atypical ductal hyperplasia. Surgical excision was planned. Eight months later she returned with a palpable lump in the left upper outer quadrant. (**B**) Left mammography was performed with a triangular skin marker taped at the site of the lump. The post biopsy marker clip was seen (arrow), but no abnormality was seen at the site of the lump on full field digital mammography (FFDM) or on digital breast tomosynthesis (not shown). (**C**) Ultrasound showed a 3.2 cm mass (arrows) and abnormal axillary nodes (not shown). Core biopsy confirmed invasive ductal carcinoma. This is an interval cancer.

There are two risks associated with dense breasts. The more important risk [with respect to screening performance] is the masking of cancers on mammograms, because they can be obscured in normal dense tissue [6,7]. Mammographic sensitivity is inversely proportional to the degree of breast density. Sensitivity for digital mammography is 79.9% overall, but almost 100% in Category A, 83.9% for Category B, 72.9% for Category C, and 50% for Category D [8]. Stated another way, as many as 50% of cancers are missed on mammograms in women with Category D. This explains why women do not all benefit equally from mammography (Figure 2).

Mortality reduction is significantly lower in women with dense breasts. Using data from the Nijmegen (Dutch) screening program, Van der Waal et al. showed 41% mortality reduction in women with fatty breasts (that they defined as <25% dense) but only 13% mortality reduction in women with dense breasts (that they defined as >25% dense) [9]. Chiu et al. showed that women with dense breasts had significantly increased breast cancer mortality (RR = 1.91) after adjusting for other risk factors [10].

The other risk for women with dense breasts is an increased risk of getting breast cancer, but the magnitude of increased risk varies, possibly in part because of the different ways of measuring density. Engmann et al. found that high breast density was the most prevalent risk factor for both premenopausal and postmenopausal women [11]. Boyd et al., using a semi-quantitative method called interactive thresholding to determine breast density found that women with dense breasts were up to six times higher risk than women of the same age with little or no density [12]. Using a five-category scale of percentage breast density, McCormack and dos Santos Silva, in a meta-analysis of 42 studies using three different density-grading methods, found increasing risk of breast cancer, with 4–6 times higher risk for women with >75% density compared to those with <5% [13]. Bertrand et al., pooling data from six studies where density was calculated by the same interactive thresholding method described by Boyd, found that women with high breast density (>51%) are twice as likely to develop breast cancer as women with average density (11–25%) [14]. It should be noted that the use of >51% most closely resembles a combination of BI-RADS Categories C and D. Using data from the Kopparberg randomized controlled trial in Sweden, Chiu et al. demonstrated increased breast cancer incidence for women with high breast density (defined as pattern IV or V by the Tabár classification) after adjusting for other risk factors [10,15].

Breast density is also a biomarker for predicting response to neo-adjuvant chemotherapy. Skarping et al. showed that premenopausal women with high mammographic density respond poorly to neoadjuvant chemotherapy [16].

Woodard et al. found that women with Category D breasts who have been treated for breast cancer had lower Oncotype DX test recurrence scores than women with Category A breasts [17]. This would seem counterintuitive, but they theorized that it might be due to “deregulation of adipose tissue signaling pathways within the breast microenvironment, leading to local aromatization of androgens to estrogens that may promote tumor recurrence.”

However, Huang et al., in a case-control study showed a higher risk of locoregional recurrence for women with Categories C and D after modified radical mastectomy [18] and Eriksson et al. found that high mammographic density (defined as >25% density) was associated with increased risk of local and locoregional recurrence compared to women with <25% density, but was not associated with distant metastasis nor survival [19]. 

Dense tissue also limits detection of recurrent breast cancer in treated women.

## 4. Density and Interval Cancers

Interval cancers (ICs) are those detected in the interval between planned screening mammograms, i.e., after a negative screening mammogram. Some ICs arise because they are rapidly growing, and were not visible on the prior mammogram but are visible on the mammogram performed at the time of diagnosis (“true IC”) [20]. Some may have been present, but not seen because of poor technique or positioning (the cancer was not included on the image), not seen because of masking by dense breast tissue, or visible in retrospect but not detected or misinterpreted by the screener (“false negative”). 

ICs are more common in younger women, women with a family history of breast cancer and women on menopausal hormone therapy. They tend to be larger and higher grade than screen-detected cancers, more likely estrogen receptor negative (ER-)and progesterone receptor negative (PR-negative), more likely mucinous and lobular histology, more likely with nodal metastases at the time of diagnosis, and with a higher proliferation index [14,19,20,21,22,23,24,25].

The interval cancer rate will vary according to the breast cancer incidence in a given population, how ICs are determined, whether initial or subsequent screening round, and the length of time between screens. Longer intervals between screening examinations lead to increased IC rates. Screening programs will have varying targets for ICs depending on their screening interval. Jurisdictions that screen every 2–3 years may accept a higher IC rate than programs that screen annually. Lehman et al. reviewed performance measures in the Breast Cancer Consortium from 2007 to 2013 and found an IC rate of 0.8/1000 within 12 months of a negative mammogram [26]. Houssami and Hunter reviewed 24 screening programs globally, most of which screen biennially, and found that 20–25% of ICs are false negative, but IC rates are lower for annual screening intervals and higher for triennial screening. IC rates ranged from 8.4 to 21.1 per 10,000 screens, with the larger proportion of the estimate occurring in year two of a two-yearly interval [22]. Seely et al. showed that in Canada, jurisdictions that offer only biennial mammograms to women with dense breasts have 63% higher interval cancer rates than those that offer annual mammography [27].

High breast density is associated with an increased risk of interval cancers [7,28,29,30]. Interval cancers are 13–31 times more likely in Category D breasts than in category A [6,30,31]. Van der Waal et al. showed that the proportion of women with dense breasts among interval cases ranged from 38.7% to 54.5%, but ranged from 20.7% to 30.5% in screen-detected cases in the Dutch screening program [9]. ICs in women with dense breasts are 2–3.5 more lethal than in women with non-dense breasts [9,32].

An important goal of breast cancer screening is to minimize ICs in women of all breast densities. Research, including artificial intelligence, is underway to determine which women are at risk of interval cancers. Several imaging modalities, including ultrasound (US), digital breast tomosynthesis (DBT), magnetic resonance imaging (MRI), contrast-enhanced mammography (CEM) and molecular breast imaging (MBI) are capable of detecting cancers missed on mammograms.

The efficacy of any imaging modality should ideally be shown by decreased mortality in RCTs. Unlike in therapeutic trials, these take decades to mature, since for lower mortality to be evident requires that there be deaths in the control group. Because of improvements in therapy, women in the control group might not die for many years, or even decades, meaning that by the time RCT results are available, the technology used in the trials may be obsolete. Kuhl and Baltzer argue that efficacy should be determined by surrogate measures such as decreased rates of ICs and advanced cancers [33]. This should be reasonable, especially given that using surrogate endpoints has been shown to underestimate benefit [34], and it would allow the introduction of life-saving technology sooner. Moreover, what really is of importance to an individual woman in the shared decision-making process regarding participation in supplemental screening is not the outcome of a trial where there was noncompliance among women in the study group, and contamination by women in the control group, but rather the outcome for women who did participate.

## 5. Ultrasound (US)

Ultrasound (US) uses sound waves to image tissue. It uses no ionizing radiation, and intravenous contrast is not required. It was used for abdominal and pelvic imaging in the 1970s and early 1980s. Breast ultrasound was attempted then with the available lower frequency transducers, and the possibility of screening with US was explored, but was unsuccessful [35,36]. With the development of high-frequency transducers in the mid 1980s, it was possible to use US in the breast: initially for cyst/solid differentiation [37], and then benign/malignant differentiation [38,39]. Since the first demonstration of US-detection of cancers missed on mammograms in 1995 [40], numerous single center studies confirmed the potential of US as a supplemental screen for women with dense breasts [41,42,43,44,45]. Summarizing these, 94% of the cancers were invasive, and 70% of them were 1 cm or smaller in size. Ninety percent were stage 0 or stage I and 90.5% of the women with sonographically-detected cancers had either heterogeneously dense or extremely dense parenchyma [46]. These studies showed a biopsy rate of 3%, and 11% of the biopsies showed malignancy. Seven percent of women had short-interval follow-up. Overall, the incremental cancer detection rate (ICDR) was 3.5 cancers/1000 screens. This increased cancer detection was of invasive cancers, so concern regarding “overdiagnosis” of DCIS did not apply.

The Avon-ACRIN (American College of Radiology) 6666 multicenter study recruited 2809 women at 21 sites, who were at increased risk and who had dense breast tissue in at least one quadrant, to have three consecutive screening examinations with mammography and breast US. The goal was to determine the ICDR of annual breast ultrasound screening, and all participants were offered breast MRI at the conclusion of the study. They found that US and MRI both detected cancers missed on mammography, at the cost of low specificity. ICDR and positive predictive value (PPV) for US was 5.3/1000 and 11% in the first year, and 3.7/1000 and 16% in the second and third years, averaging 4.3/1000 and 14% overall. Of the cancers seen only on US, 94% were invasive with a median size of 10 mm, and 96% of those staged were node negative. MRI detected an additional 14.7/1000 after negative US and mammography [47]. 

False alarms, i.e., women recalled for additional tests at screening but found to be benign after diagnostic workup, cause anxiety for women. Those that are determined to be “probably benign,” for which surveillance can be recommended instead of biopsy, incur costs and use appointment slots that could be used for more urgent cases. Barr et al. investigated the outcomes of 745 BI-RADS 3 masses in the Avon ACRIN 6666 trial, and found 6 cancers (0.8%). Only one of the cancers had suspicious changes at 6-month follow-up, so it is reasonable to drop the necessity of a 6-month US and recommend a 1-year diagnostic follow-up for BI-RADS category 3 lesions detected at screening US [48].

In 2009, Connecticut was the first state to enact breast density notification legislation, and to perform high volume supplemental US screening. Hooley reported the initial experience with technologist-performed US, which showed ICDR of 3.2 cancers/1000 prevalence screens. PPV was 6.5% [49]. At RSNA 2016, Philpotts et al. reported on the outcomes during their 5th-year and showed ICDR of 2.6 cancers/1000 screens, with a much-improved PPV of 25% [50]. Elsewhere in Connecticut, Weigert and Steenbergen reported on their first year of screening US: ICDR was 3.25 cancers/1000 screens with a PPV of 6.7% [51]. Their 4th year showed stable ICDR of 3.3 cancers/1000 and much-improved PPV 20.1% [52]. Currently, there is insurance coverage for supplemental ultrasound screening in 12 states and the District of Columbia, but the laws vary by state, and out-of-pocket costs may apply [53]. 

**Figure 3:**
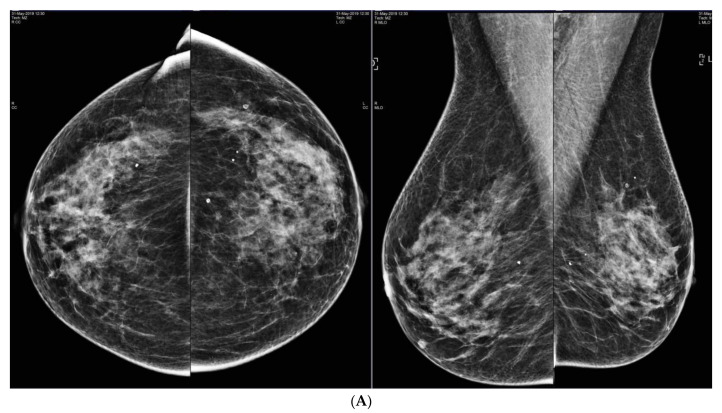

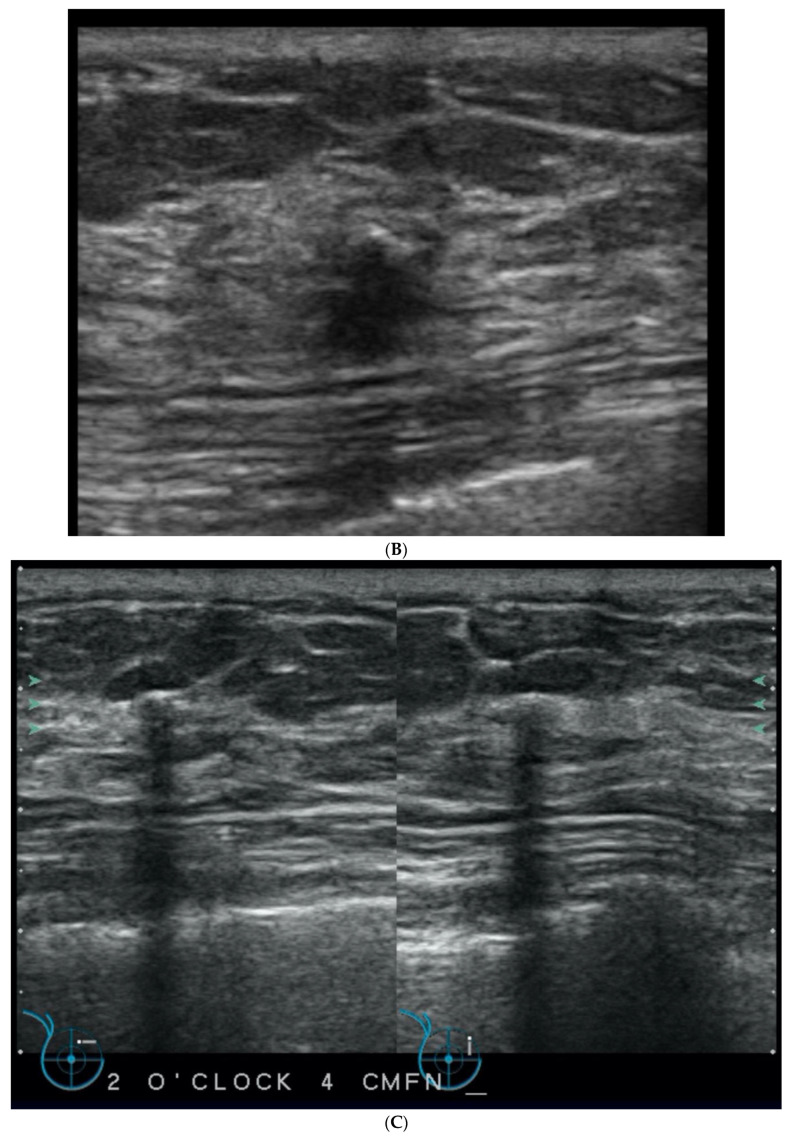
(**A**) Negative screening mammogram in a 73-year-old woman with category C density. Screening ultrasound showed masses in (**B**) left and (**C**) right breasts. Tomosynthesis is not part of the screening mammography program, but was performed after masses were found on US, but was also negative (not shown). Mass on left showed invasive lobular carcinoma on core biopsy. Mass on right showed atypical lobular hyperplasia.

Breast density notification is also underway in in Canada. In response to patient advocacy, the first of several provinces started notifying all women of their breast density on their screening mammography reports in 2018 [54]. Coverage by public health insurance followed in early 2019 [55]. Women in British Columbia with category C and D densities and no other risk factors may attend supplemental US screening, with a referral from their physician or nurse practitioner. Wu and Warren reported on the first year of screening US after insurance coverage started, and showed ICDR of 7/1000, with average tumor size of 9 mm (all node negative), biopsy rate of 1.3% and PPV of 42% (Figure 3). Importantly, 40% of the cancers were found in women with no risk factors other than dense breasts [56]. Sixty percent of the cancers were found in women with category C density [57]. This is relevant, as some have suggested that supplemental screening should be rationed to only the smaller percentage of women with extremely dense breasts.

Acknowledging the importance of increasing ICDRs and reducing ICs, Corsetti et al. reported on a retrospective cohort of 8865 women studied from 2001–2006. Women with non-dense breasts had mammography alone, and women with dense breasts had mammography and US. After one year, CDR was 6.3/1000 screens in the mammogram group, and 8.3/1000 screens in the mammogram plus US group. Overall, there was no significant difference in the IC rate: 1.0/1000 in women with non-dense breasts, and 1.1/1000 in women with dense breasts. The ICs were early stage (in situ or small invasive) cancers, and almost all were node-negative. They admitted that cancers not seen on mammography may or may not progress to become ICs, and that reducing ICs might not reduce mortality, and stated the importance of an RCT [58].

An RCT of screening US, the Japan Strategic Anti-cancer Randomized Trial (J-START), is underway in Japan. From July 2007 to March 2011, 72,998 women aged 40–49 of all breast densities were enrolled at 42 study sites and divided into 2 equal groups, one having mammography alone (control group), and the other having mammography plus US (intervention group) annually for 2 years. In a preliminary publication, sensitivity was significantly higher in the intervention group than in the control group (91·1% vs. 77·0%; *p* = 0.0004). Specificity was significantly lower (87·7% vs. 91·4%; *p* < 0.0001). More cancers were detected in the intervention group (184 vs. 117; *p* = 0.0003) and were more frequently stage 0 and I (144 vs. 79; *p* = 0.0194). Fewer ICs were detected in the intervention group 18 vs. 35; (*p* = 0.034) [59]. Further follow-up is anticipated in the near future.

Harada-Shoji et al. did a secondary analysis of the J-START trial to determine the effect of breast density on outcome of adding ultrasound to mammography. The unexpected finding that women with non-dense breasts benefitted equally to women with dense breasts, indicates that there is room for improvement in screening women of all densities in this younger age group [60]. As Kuhl points out, it is noteworthy that there was a greater proportion of women with dense breasts in the study compared with Western women: 68% vs. 43% [4], and the peak incidence of breast cancer in Japan is much younger than in women in the USA or Europe: aged 40–49 vs. 60–69 [61,62]. So not surprisingly, Kuhl sees this study as a reminder that there is a need for research on improved screening strategies for women of all breast densities [62]. Her work in 2017 showed that supplemental MRI screening in average-risk women of all breast densities detected 22.6 cancers/1000 prevalent screens and 6.9/1000 incident screens with no interval cancers, and the MRI-detected cancers were high-grade in 41.7% of cases at prevalence screening and 46.0% of cases at incidence screening [63].

Ohnuki et al. addressed the lower specificity in the intervention group that arose in the J-START trial, as a result of further examination being required in all positive cases classified by either mammography or ultrasound. They developed an overall assessment system of combined mammography and adjunctive ultrasound for breast cancer screening which lowered recall rates by 16–53% [64]. For example: if mammography showed a mass requiring recall, but concurrent US showed a benign lesion, then recall could be avoided.

Initially performed as a hand-held examination, automated US equipment using large footprint transducers was developed, and tested in the Somo-Insight multicenter trial, where 15,318 average-risk women with Category C and D density were recruited. The ICDR was 1.9 cancers/1000 women, but with an increased recall rate over mammography of 285/1000 [65].

Kelly et al. used a device with a small-footprint hand-held type transducer attached to an automated articulated arm. The images were acquired with the transducer moved in overlapping vertical rows and were viewed in cine loops. The ICDR was 3.6 cancers/1000; recall rate was 7.2%; PPV 38% [66].

Berg and Vourtsis reviewed ICDR in hand-held and automated breast ultrasound, and reported similar ICDRs averaging 2.1–2.7/1000 for physician- and technologist-performed examinations, but the ranges are wide. This may be due to the differences in the time periods encompassed. The averages using hand-held included studies date from 1995, so the earlier studies were performed with more primitive technology. The automated studies were from 2010–2018, so used more advanced technology [67].

Observational data showing surrogate markers of tumor size and lymph node status indicates that supplemental US finds the same kinds of cancers that led to decreased mortality in the RCTs of mammography. It has been shown that using surrogate markers underestimates benefits [34]. Nevertheless, task forces in Canada and the USA do not recommend supplemental screening for women with dense breasts, because they decline to consider data other than RCTs. This underscores the value of the J-START trial.

Supplemental US screening is available in some locations in Canada [68], the USA [69], and Europe [70]. Currently, there is insurance coverage in 12 American states and the District of Columbia, but the details vary by state [53]. Shared, informed decision-making is recommended to ensure that women are aware of and prepared for possible recall. The data used for shared decision-making should include all available information, including observational studies. In France and Austria supplemental US screening is part of organized screening programs [70]. 

Whether to perform supplemental ultrasound concurrently with screening mammography, or alternating with it should take patient convenience into account, and Ohnuki’s demonstration of improved specificity is also a consideration [65]. However, where screening mammography is performed only biennially, alternating the ultrasound with mammography (each modality every 2 years) allows more frequent screening, and the opportunity for earlier detection.

## 6. Digital Breast Tomosynthesis (DBT)

DBT is a quasi-three-dimensional technique, where multiple low-dose images are obtained over a range of angles, and reconstructed in slices [71]. It received United States Food and Drug Administration (FDA) approval in 2011. Initially, DBT was performed in addition to digital mammography. Increasingly, if is performed with “synthetic two-dimensional (2D),” which reduces dose by half [72].

**Figure 4:**
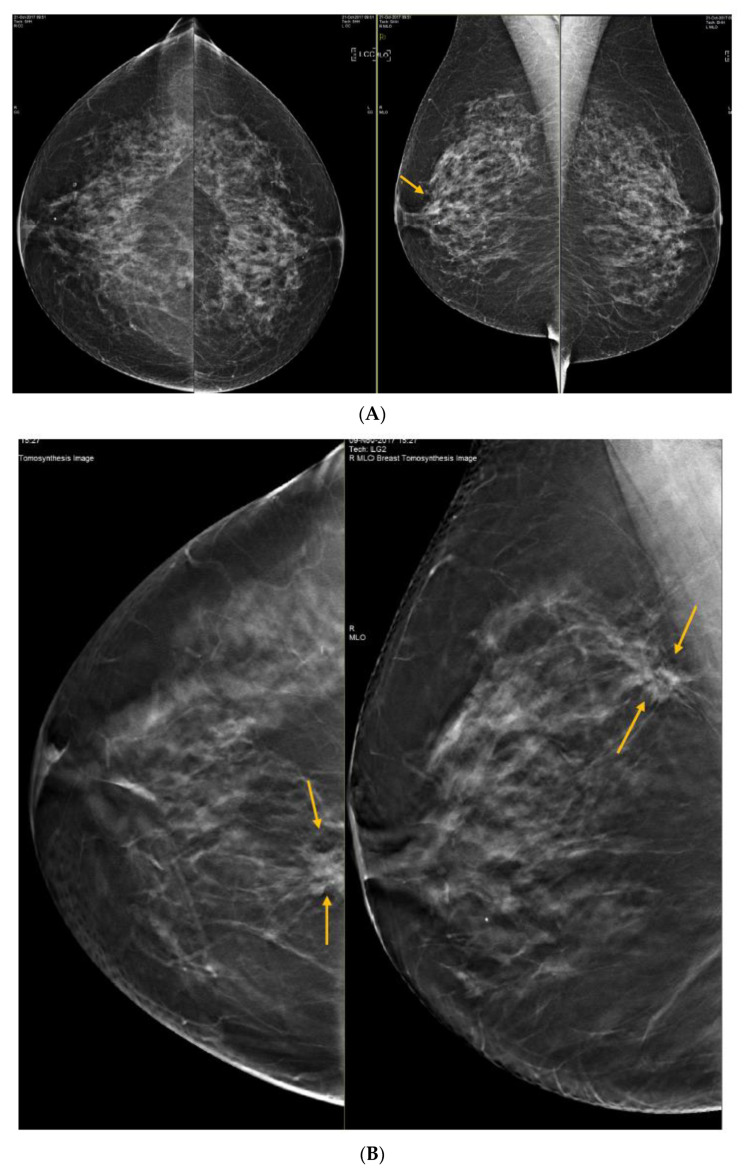

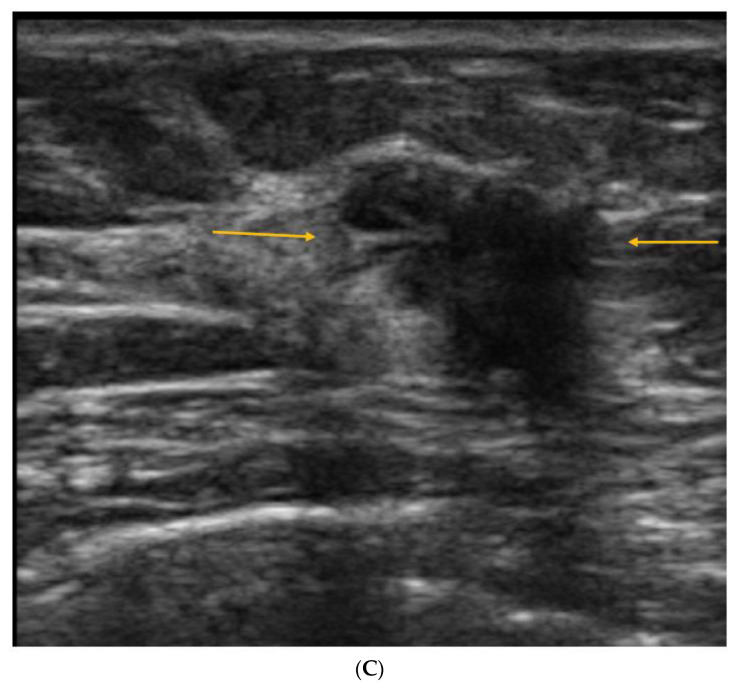
Illustration of DBT resolving an asymmetry seen at 2D screening mammography in the right retroareolar region, and unmasking a cancer in the right upper inner quadrant, not seen on the 2D mammogram. (**A**) This 58-year-old woman had a questionable asymmetry seen on her 2D screening mammogram on the right mediolateral oblique (MLO) view in the retroareolar region (arrow). (**B**) DBT was performed and the asymmetry was shown to be normal tissue, but a spiculated mass was seen in the right upper inner quadrant posteriorly (arrows). (**C**) A correlate was seen on US (arrows), for which histology showed invasive ductal carcinoma.

DBT increases cancer detection by reducing superimposition, thereby unmasking abnormalities that are obscured by overlapping tissue [73,74,75], and these are maintained beyond the prevalence round [76]. Invasive lobular cancers in particular, are more conspicuous because of increased visibility of spiculation and architectural distortion [73,77]. The additional cancers detected are invasive, not DCIS, which minimizes “overdiagnosis”. DBT reduces recalls by confirming that many apparent abnormalities are artifactual, caused by summation of normal structures [78]. Figure 4 shows an example of a women screened with only 2D digital mammography, where an asymmetry was seen on one view only. Her diagnostic examination was performed with DBT, which confirmed the screening finding to be summation artifact, but also unmasked a cancer that was missed on the 2D screening study. 

DBT is widely used in opportunistic screening in the USA. As of 1March 2022, 82% of facilities had DBT units and 45% of all accredited units were DBT in the United States [79]. There is legislation in 17 states and the District of Columbia requiring insurance coverage for DBT screening, but the details of coverage vary by state [53]. However, beyond state law mandates for tomosynthesis coverage, nearly every major insurer in the United States now covers DBT, although out of pocket costs may apply.

DBT is not used in any of the organized screening programs in Canada, but is available for women participating in the TMIST trial in six Canadian cities [80]. It is used in some European countries either in organized screening or opportunistically [70]. The ongoing TMIST trial aims to determine whether DBT reduces advanced cancers, defined as: those with distant metastases, positive lymph nodes, or invasive cancer of greater than 20 mm. Invasive cancer greater than 10 mm but less than 20 mm in size are considered advanced cancer if they are either triple negative or human epidermal growth factor receptor 2 positive (HER2+) [81]. So far, ICs have not shown to be reduced [82,83].

**Figure 5:**
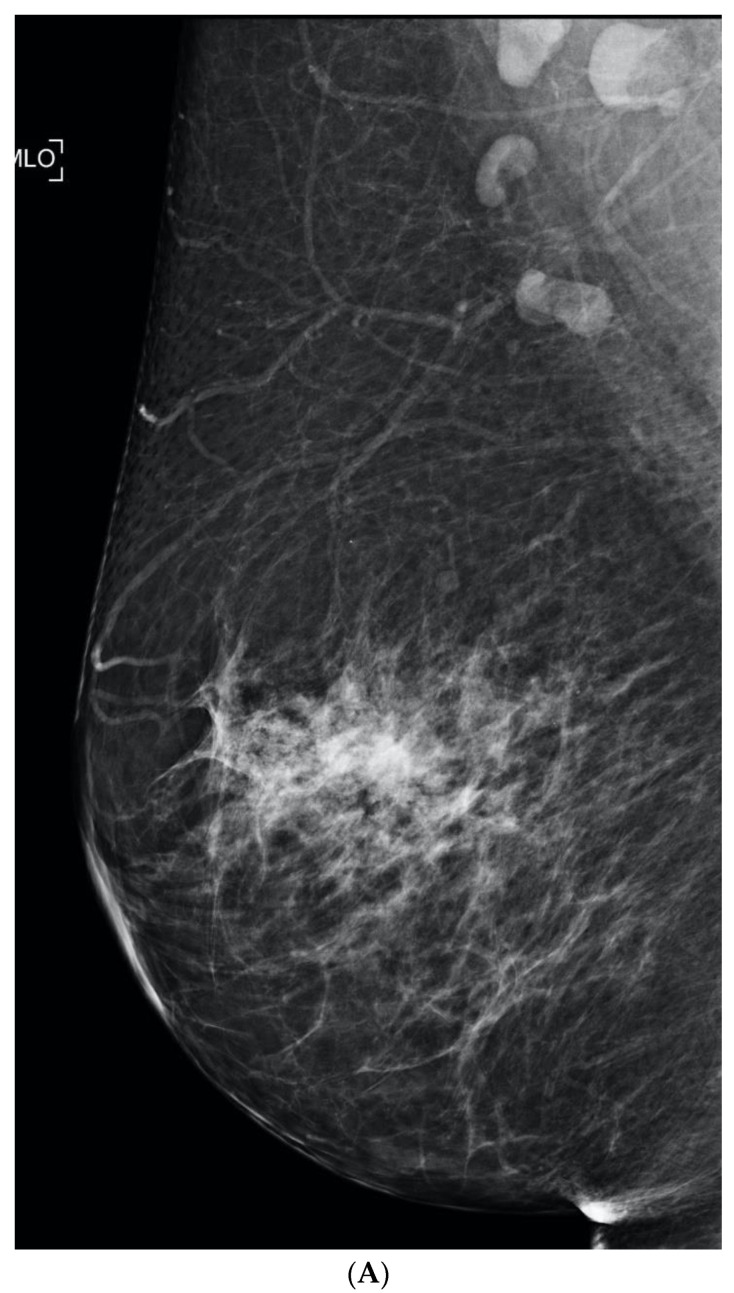

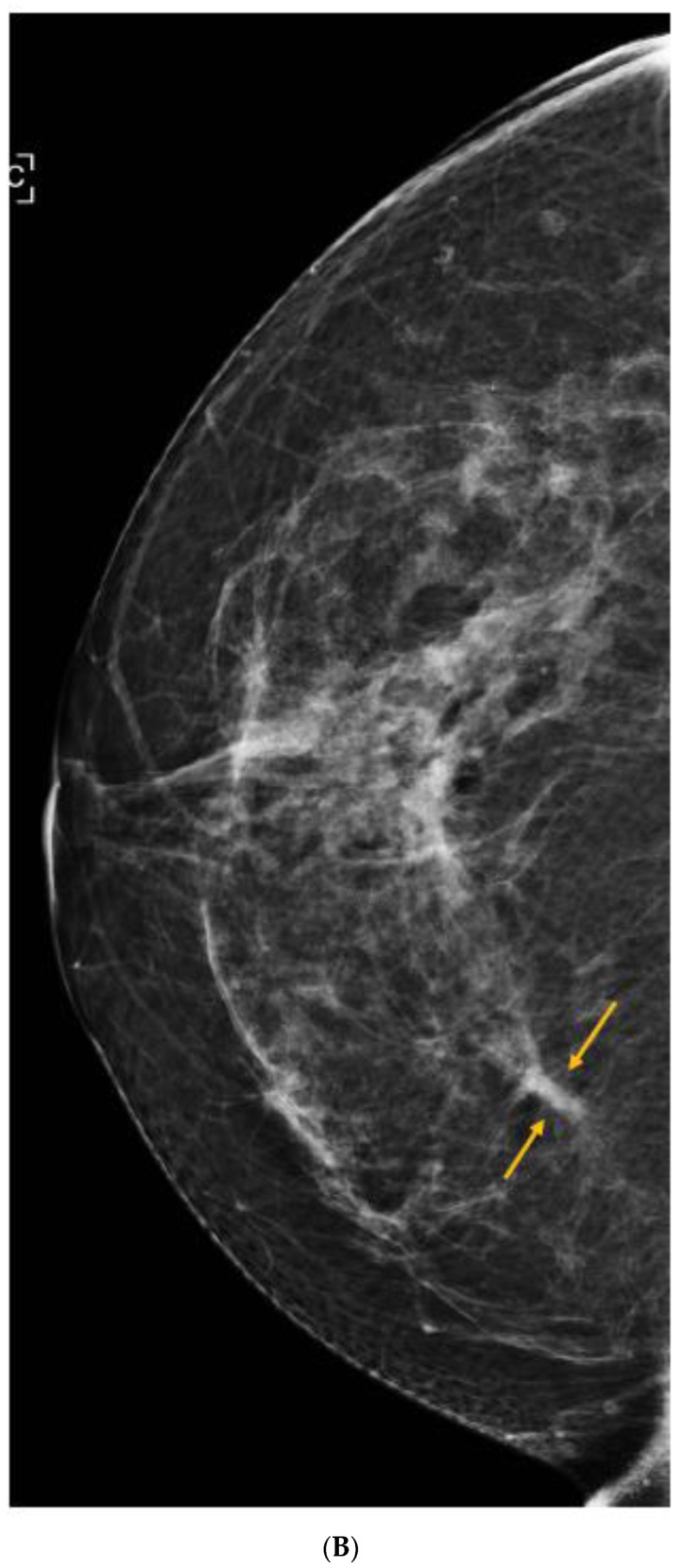

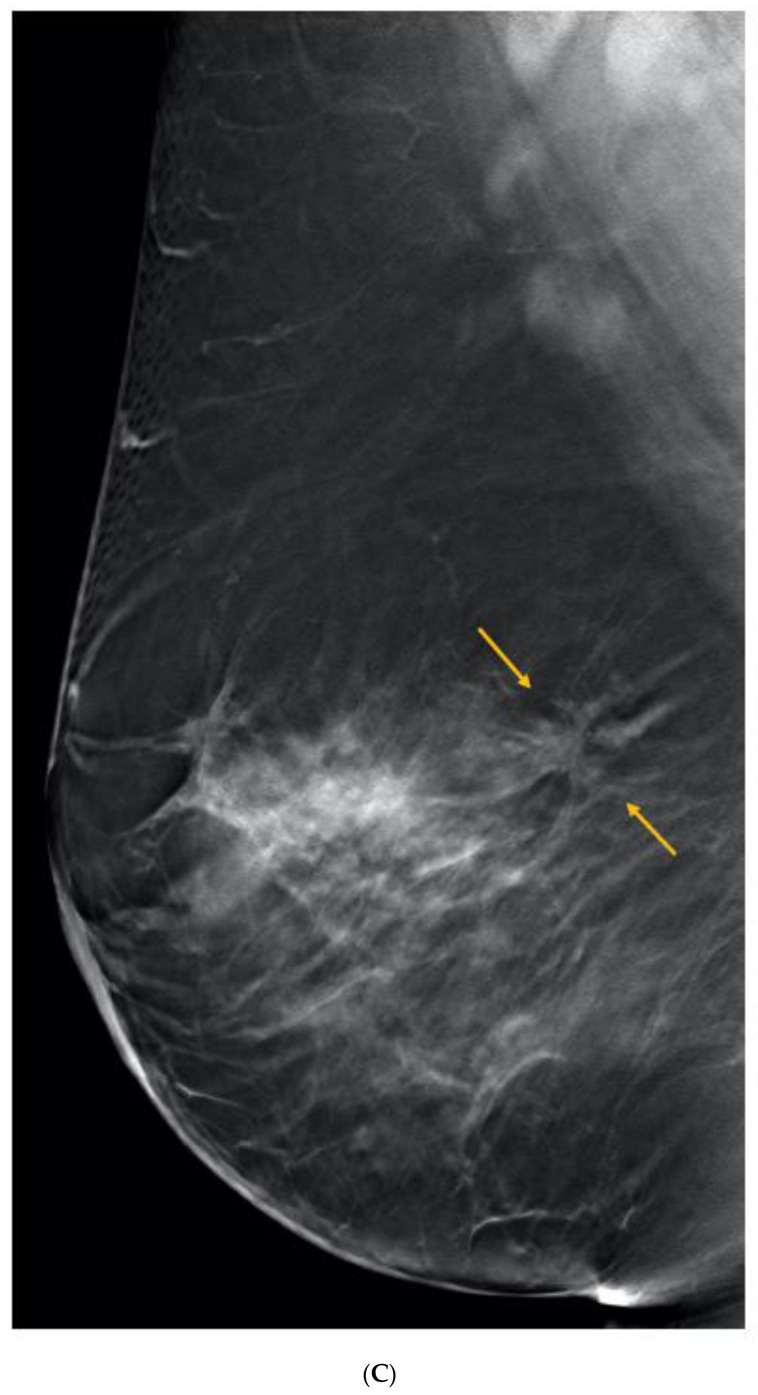

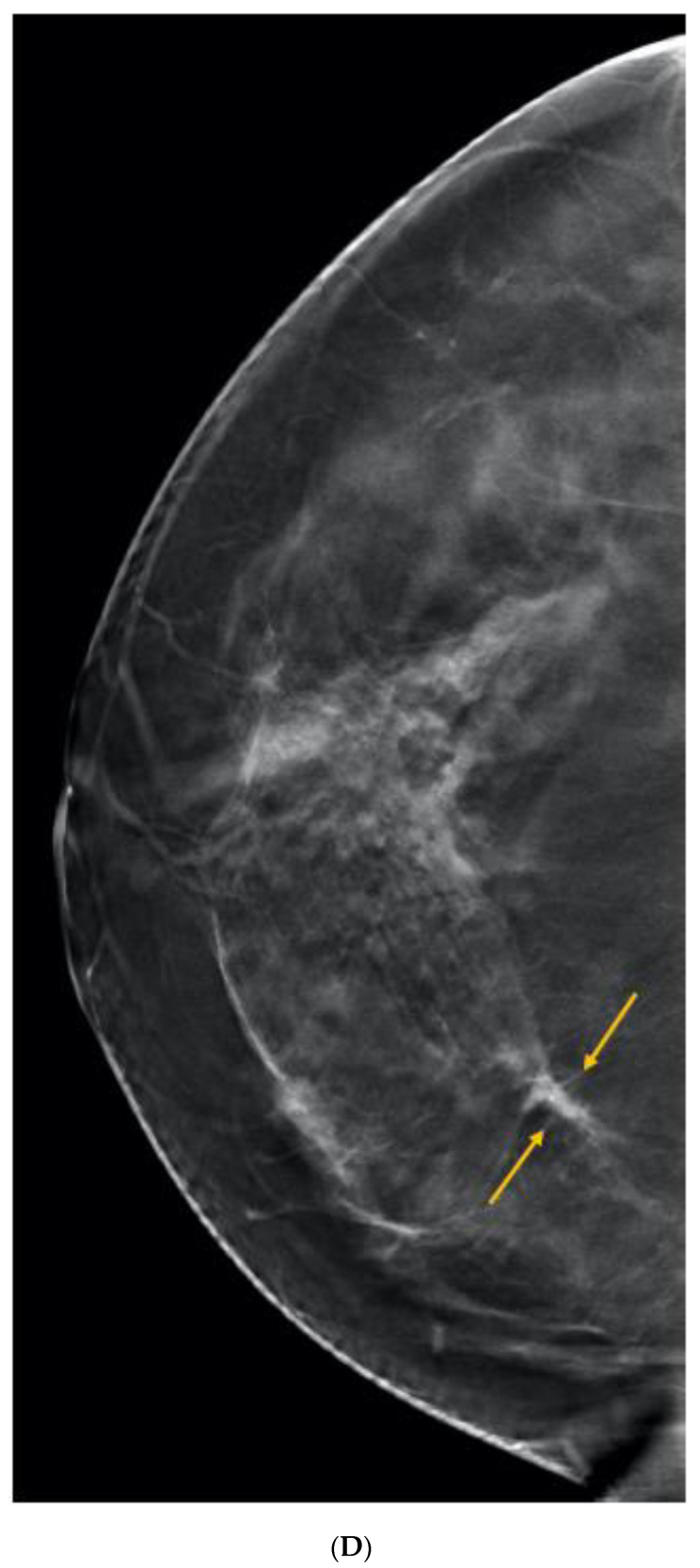

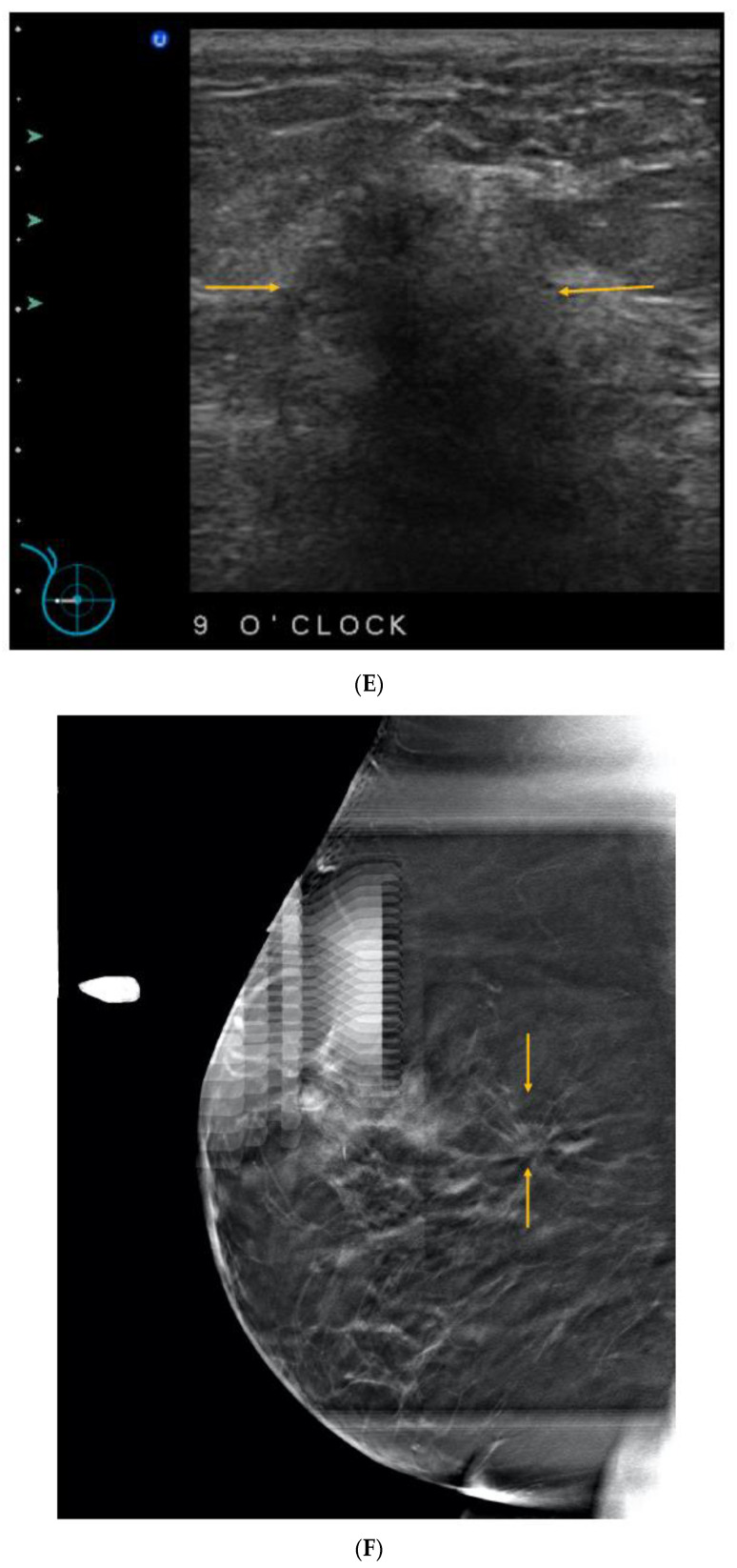
Fifty-eight-year-old woman with 2 cancers: one seen on DBT but not visible with US, and one seen on US but not on DBT. She presented for screening with DBT. (**A**) Right 2D MLO view was negative, but asymmetry was seen (**B**) medially on the craniocaudal (CC) view. DBT (**C**) MLO and (**D**) CC views show architectural distortion (arrows) in the upper inner quadrant. US was negative in the upper inner quadrant (no image), but showed a suspicious mass in the 9 o’clock position (arrows in (**E**). (**F**) The upper inner quadrant mass was biopsied with DBT-guidance (scout view) and was an invasive lobular carcinoma. The 9 o’clock mass was biopsied with US-guidance and was an invasive ductal carcinoma.

**Figure 6:**
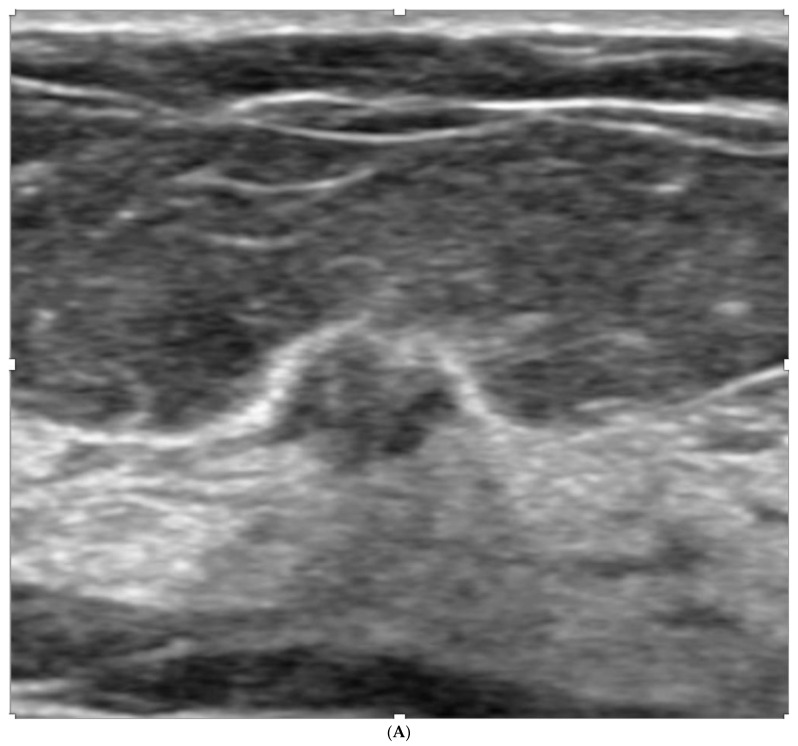

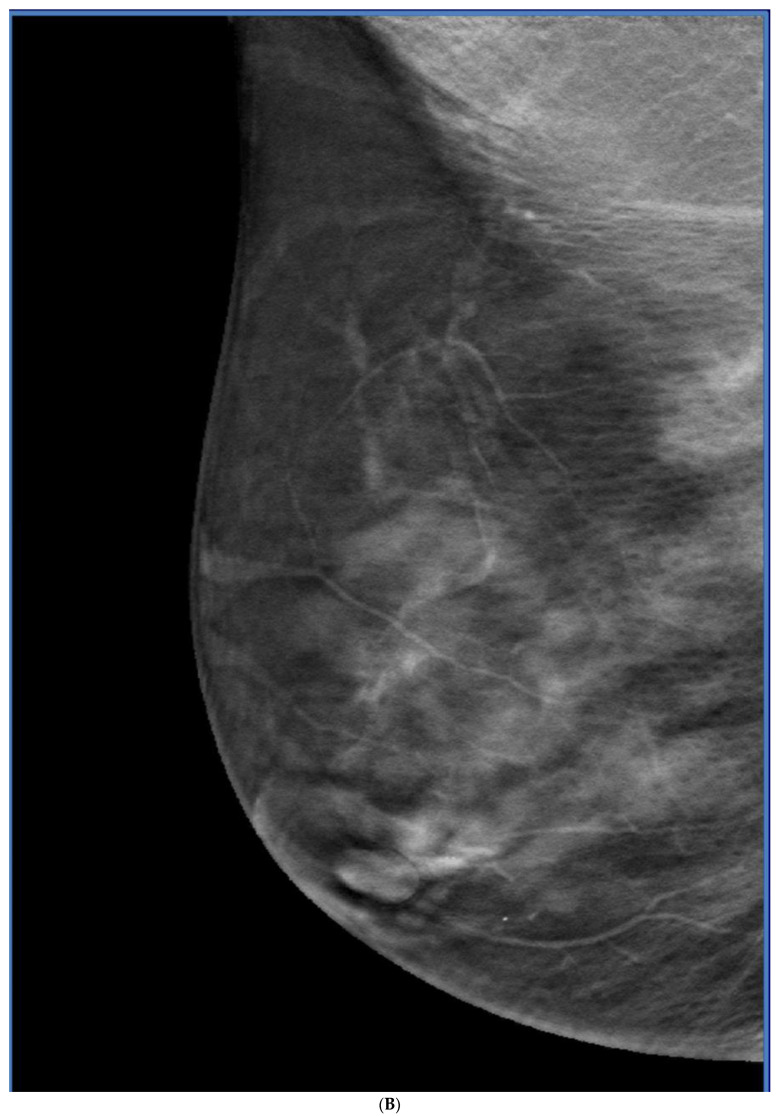

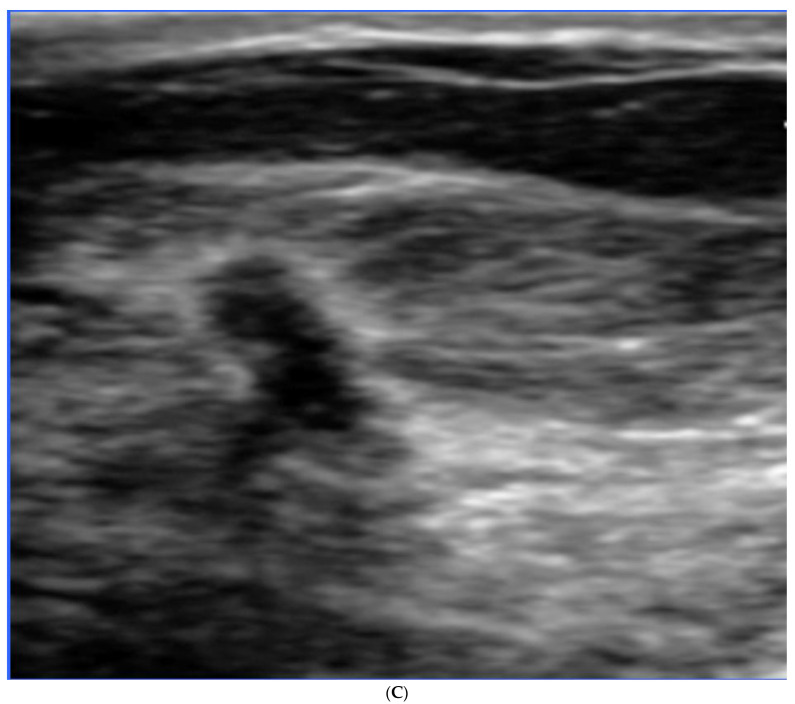

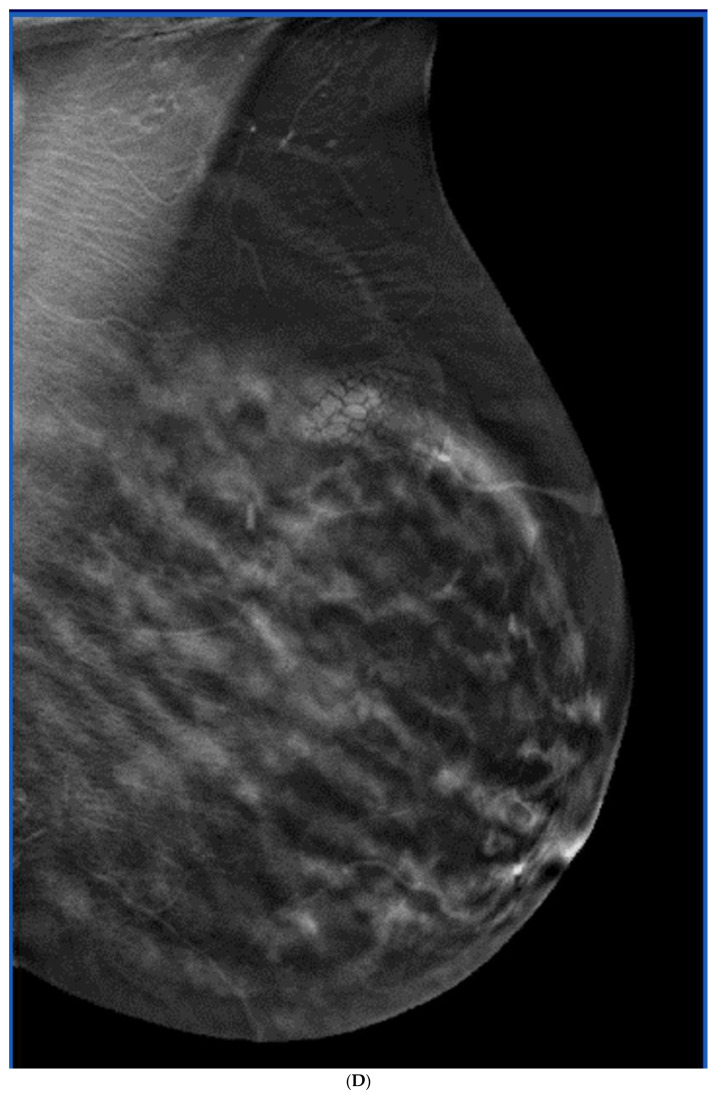

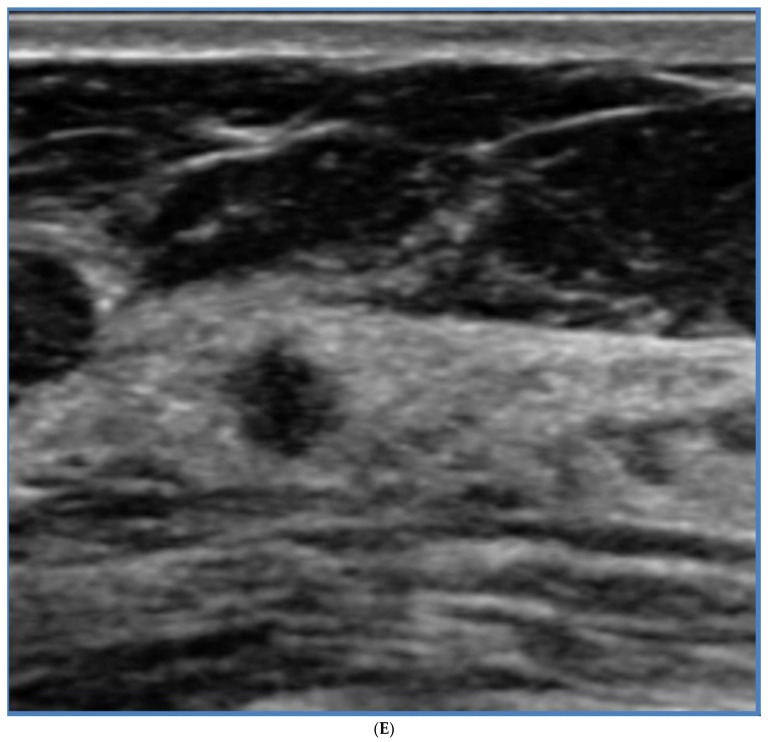

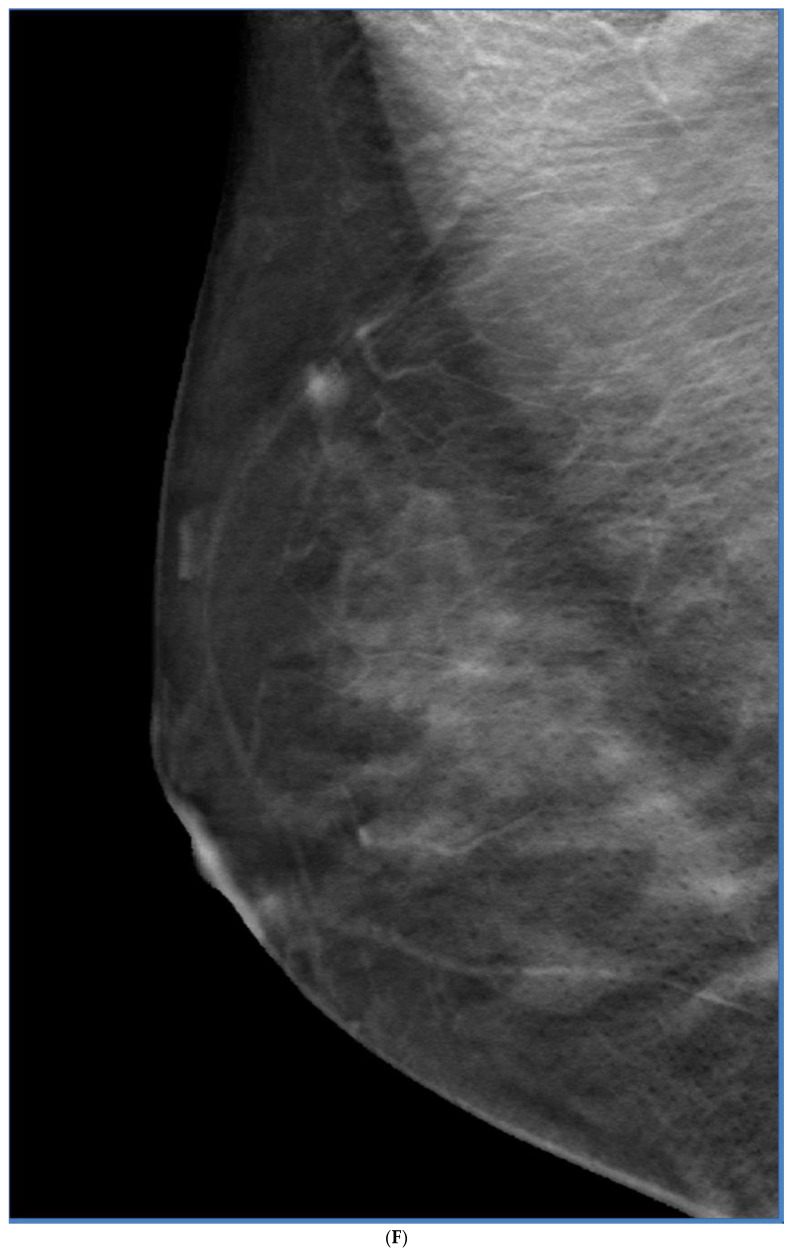
Courtesy of Dr. Regina Hooley. Three cancers seen on screening US, that were missed on DBT: Mass #1 (**A**) US and (**B**) DBT; Mass #2 (**C**) US and (**D**) DBT; Mass #3 (**E**) US and (**F**) DBT.

Rafferty et al. studied the effectiveness of DBT according to breast density, and documented that it is most beneficial in heterogeneously dense breasts, but adds no significant cancer detection in extremely dense breasts [84]. So DBT does not obviate the need for supplemental screening for women with dense breasts (see Figure 2). The ASTOUND trial prospectively compared DBT and US in mammographically-negative dense breasts. US detected almost twice as many additional cancers as DBT at a similar recall rate [85,86]. Figure 5 shows a cancer seen only on DBT, even in a relatively fatty area in a category B breast. US was performed to determine whether a correlate would allow an US-guided core biopsy. The DBT-detected cancer was not seen on the US, and was biopsied with DBT-guidance, but the US revealed a second cancer that was not seen on the DBT, and it was biopsied with US-guidance. Figure 6 shows 3 cancers missed on DBT, but seen with US.

There is significant disparity in breast cancer screening. Although black women have a lower incidence of breast cancer compared with white women, they have higher breast cancer mortality [87,88]. This may be due, in part, because on average, black women have higher breast density than white women [89] and are diagnosed with advanced disease more often [87,88,90]. TMIST will compare how breast density affects the detection of advanced cancer between digital mammography and DBT [81]. 

DBT has been called “a better mammogram”, and this claim is borne out in its improved sensitivity and specificity compared to digital mammography. It does not, however bring equity in breast cancer screening in women with dense breasts.

## 7. Magnetic Resonance Imaging (MRI)

Contrast-enhanced MRI (CE MRI) is the most sensitive imaging modality for breast cancer detection, even more so than combined mammography and US [91,92], and when women are being screened with MRI, US finds no additional cancers [92]. Importantly, whereas mammography and DBT are relatively more sensitive to less aggressive cancers, and less sensitive to biologically aggressive cancers, MRI is the opposite. It is arguable that MRI’s lower sensitivity to low-grade DCIS is a good thing, leading to reduced “overdiagnosis.” Another advantage compared to other modalities is that axillary lymph nodes are included in the scan field. CE MRI uses no ionizing radiation but requires intravenous injection of Gadolinium, which can be classified as linear or macrocyclic. Gadolinium has been shown to accumulate in bone [93,94], skin [95], solid organs [96], and brain tissue [97], albeit with no known long-term effects at the time of writing. Macrocyclic gadolinium is more stable and less likely to accumulate in organs [98].

CE MRI has been shown to be helpful in staging newly-diagnosed breast cancer, guiding surgical planning, and assessing treatment response during/after neoadjuvant chemotherapy [99,100]. 

**Figure 7:**
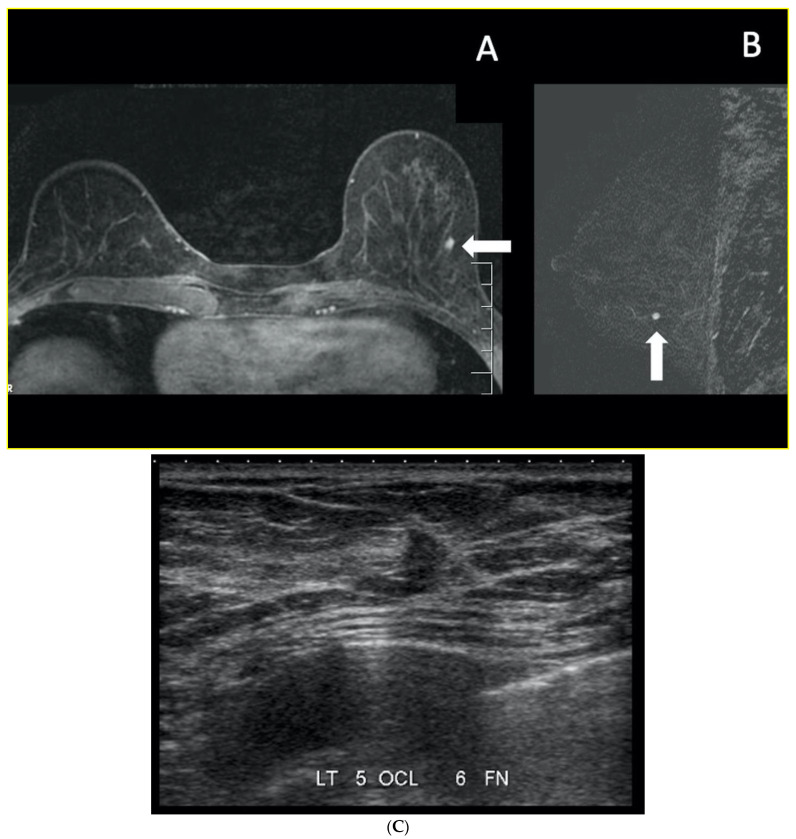
Courtesy of Dr. Anabel Scaranelo. High-risk screening with breast MRI in a 52-year-old woman with a family history of breast cancer in her sister. Screening mammography (not shown) was negative, with category C density. Focus of enhancement (arrow) in the lower outer quadrant of the left breast, (**A**) axial and (**B**) sagittal T1-weighted 3D-gradient echo with fat saturation 2nd minute post contrast. (**C**) The patient was recalled for a second look ultrasound that showed correlation with a suspicious subcentimeter mass lesion at 5 o’clock (arrows). An ultrasound-guided core needle showed grade 2 invasive ductal carcinoma.

Screening MRI was first recommended for women with a lifetime risk ≥ 20% by the American Cancer Society in 2007 [100]. These guidelines were expanded over time, and the American College of Radiology now recommends MRI for women with genetics-based increased risk (and their untested first-degree relatives), with a calculated lifetime risk of 20% or more, women with a history of chest or mantle radiation therapy at a young age, women with dense tissue who have been treated for breast cancer, or those diagnosed by age 50. They recommend consideration of MRI surveillance for women diagnosed with breast cancer or atypia at biopsy, especially if they have other risk factors are. They suggest that ultrasound be considered for those who qualify for but cannot undergo MRI. They recommend that all women, especially black women and those of Ashkenazi Jewish descent, should be evaluated for breast cancer risk no later than age 30, to identify those at higher risk so that they can benefit from supplemental screening [101]. Figure 7 shows a tiny cancer detected on MRI, and subsequently on directed US, in a 52-year-old high risk patient, whose 2D screening mammogram showed BI-RADS C density and was negative. MRI for screening women at high risk is standard in North America. In the USA, there is currently legislation requiring insurance coverage of MRI screening in 10 states and the District of Columbia, although details vary by state. Some specify MRI for high-risk, independent of breast density. Out-of-pocket costs may apply [53].

There is also promising research into the use of MRI for average-risk women with extremely dense breasts. The DENSE trial [102] yielded 16.5 prevalent and 5.9 incident cancers per thousand in women with category D, and ICs were reduced from 4.9/1000 to 0.8/1000 in women who actually had MRI. Kuhl studied average-risk women of all densities, whose conventional screening (mammography +/− US) was negative, and found an ICDR of 22.6 cancers/1000 in the prevalence screen, and 6.9/1000 in the incidence screens. Of the 61 cancers detected over the course of the study (41 invasive, 20 DCIS), 60 were detected only with MRI, and the other was detected with all 3 modalities; no cancer was found with mammography and ultrasound only [63]. The term “prevalence screen” is usually used in the context of a woman’s first screening examination, but in that study it refers to the first MRI, since most of the subjects had had conventional screening examinations prior to joining the study. After their last MRI, women were followed for an additional 2 years, and importantly, there were no interval cancers during that time. Reduced interval cancers in both trials suggest future mortality reduction. Of the 61 cancers, 26 (43%) were high-grade, including 46% at incident screening. Twenty of the cancers (33%) were estrogen- and progesterone receptor–negative, so biologically significant, and not over diagnosed.

Mango performed a Monte Carlo simulation cost–benefit analysis of screening average-risk women with triennial MRI compared to annual mammography beginning at age 40 over 30 years. It was based on Kuhl’s study from 2017 which used full-protocol MRI [63]. Using a cost per MRI examination of $549.71, MRI screening is more cost-effective than mammography screening in 24 years. If the cost per MRI is $400, MRI became cost-effective compared to mammography screening in less than 6 years, with over a 22% cost reduction relative to mammography screening in 12 years and reaching a 38% reduction in 30 years [103]. It is not known whether the analysis would have been even more favorable had the model been based on abbreviated MRI, or whether the $400 cost is applicable to it, since at the time of writing, there is no CPT code for abbreviated MRI.

Geuzinge used microsimulation to study the cost-effectiveness of screening MRI for women aged 50–75 in the DENSE trial, taking into consideration numbers of breast cancers, life-years, quality-adjusted life-years (QALYs), breast cancer deaths, and “overdiagnosis,” and found that MRI screening for women with extremely dense breasts is cost-effective at 3–4-year intervals compared with biennial mammography [104]. The DENSE trial used full-protocol MRI, and it is not known whether the outcome of the microsimulation would have been even more favorable had abbreviated MRI been studied.

As a result of the favorable metrics in these studies, the European Society of Breast Imaging (EUSOBI) released new recommendations in March 2022 [105]. These include informing women of their breast density, and offering women aged 50–74 with extremely dense breasts supplemental MRI screening every 2–4 years, through a shared decision-making process. They suggest that starting MRI could be adopted at a younger age, if screening is performed younger than age 50. They acknowledge that in areas where access to Breast MRI is limited, that ultrasound in combination with mammography may be used as an alternative.

Not all women accept or tolerate MRI, however. In the Avon-ACRIN 6666 trial, 42% of women offered MRI declined for a variety of reasons including claustrophobia, time constraints, financial concerns and fear of contrast injection [106]. In the DENSE trial, only 59% of the women randomized to the MRI group accepted the invitation [102]. MRI-incompatible pacemakers or cochlear implants are also potential contraindications [107]. Other contraindications include pregnancy and some spinal fixation hardware [108].

Abbreviated MRI (AB-MRI), first introduced by Kuhl et al. in 2014 [109] will reduce cost and improve access to MRI, by reducing both magnet time and interpretation time. It might also be more tolerable for women with claustrophobia. It has been shown to have equivalent diagnostic accuracy to full protocol MRI [110]. Its proven efficiency is established for screening average-risk women at all densities at reduced cost [63]. 

In the EA1141 study Comstock et al. are comparing AB-MRI with DBT screening in average-risk women with Category C and D densities. The 2020 publication includes data from the baseline studies and one-year follow-up, and additional studies will be obtained for three more years. The invasive cancer detection rate was 11.8/1000 women for abbreviated breast MRI vs. 4.8/1000 women for DBT, and there were no interval cancers [111].

Patel et al. have summarized the current AB-MRI protocols, and the inclusion of ultrafast imaging to provide kinetic information [112]. 

Because of the demonstrated minimal or zero interval cancer rate, MRI screening can be conducted at longer intervals. MRI could one day replace screening mammography but for the requirement of intravenous gadolinium-based contrast, and the limited number of scanners compared to mammography equipment. There are circumstances when requiring IV gadolinium contrast should not be a deterrent. For high-risk women, the benefits of MRI screening outweigh any potential risks of Gadolinium. It may be some time before we know if this also applies for average-risk women. The alternative functional modalities that potentially have equivalent sensitivity are discussed below. 

Diffusion-weighted MRI (DW MRI) is an MRI technique that does not use IV contrast, but measures endogenous water movement within tissue. It has been studied as a supplement to contrast-enhanced MRI to improve differentiation of benign from malignant lesions, and although the combination of DW and CE MRI yielded the best performance, DW MRI alone was comparable to CE MRI for differentiating known suspicious lesions. For screening, its sensitivity has been shown to be intermediate between CE MRI and mammography. DW MRI may be superior to US at detecting mammographically occult cancer, but detects larger cancers than CE MRI. In a study by O’Flynn, the mean size of breast cancers on DW MRI was 29.5 mm [113]. Amornsiripanitch predicts improved performance with optimal acquisition and interpretation protocols, but stresses that standardized approaches in larger patient cohorts are essential prior to widespread implementation [114]. It remains to be seen whether DW MRI can play a role in screening average-risk women, and compete with AB-MRI and contrast-enhanced mammography.

Diffusion Tensor Imaging is a variation of DW MRI with the potential to increase specificity without losing sensitivity [115].

Another potential future application of MRI is the MRI fingerprinting technique, which calculates simultaneous and volumetric T1 and T2 relaxation times for breast tissues, with the goal of being able to distinguish normal tissue from diseased tissue. It can be performed without or with contrast, but its current spatial resolution is lower than that of CE MRI, so whether it ultimately finds a role in screening remains to be seen [116].

An alternate form of non-contrast MRI called luminal water imaging is currently being studied in prostate cancer. It compares favorably with CE MRI and DW MRI [117]. It may be applicable in breast in future, but is not currently the subject of research [118].

## 8. Contrast-Enhanced Mammography (CEM)

CEM, also known as “contrast-enhanced digital mammography”, “contrast-enhanced spectral mammography”, or contrast-enhanced dual-energy mammography,” is an x-ray subtraction technique. 

Iodinated intravenous contrast (the same contrast used for computed tomography (CT) scans is administered, usually via a power injector. After about 2 min, the patient is positioned as for a screening mammogram, and the usual four images are obtained. Further detail about procedure for intravenous (IV) administration and imaging protocol is available from recent reviews [119,120]. At each position, two exposures are obtained simultaneously: One low-energy: 28–33 kilovoltage peak (kVp) and one high energy: 45–49 kVp. These are chosen to be below and above the K-edge of iodine. The low-energy image shows breast tissue and is diagnostically equivalent to a routine digital mammogram image, even though the tissue contains iodine [121]. In a study comparing low-energy with 2D images of 40 biopsy-proven cancers, Konstantopoulos et al. found that cancers may be better visualized on the low energy images compared with the 2D digital mammogram [122].

**Figure 8:**
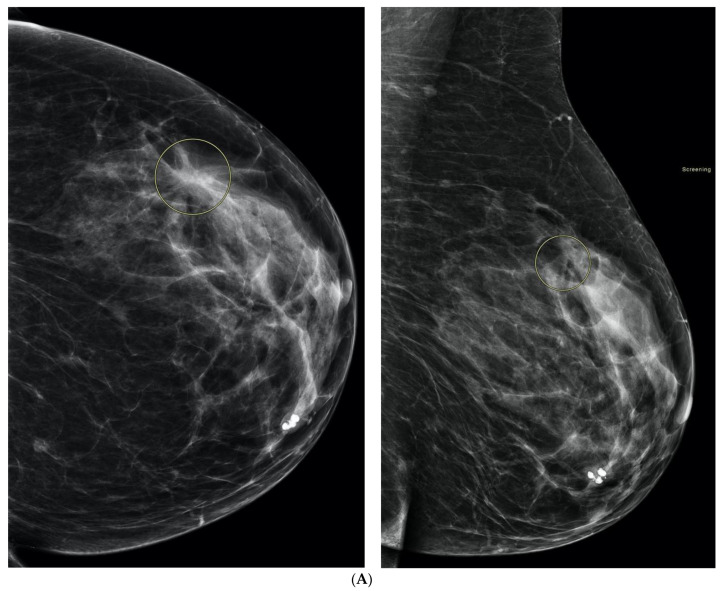

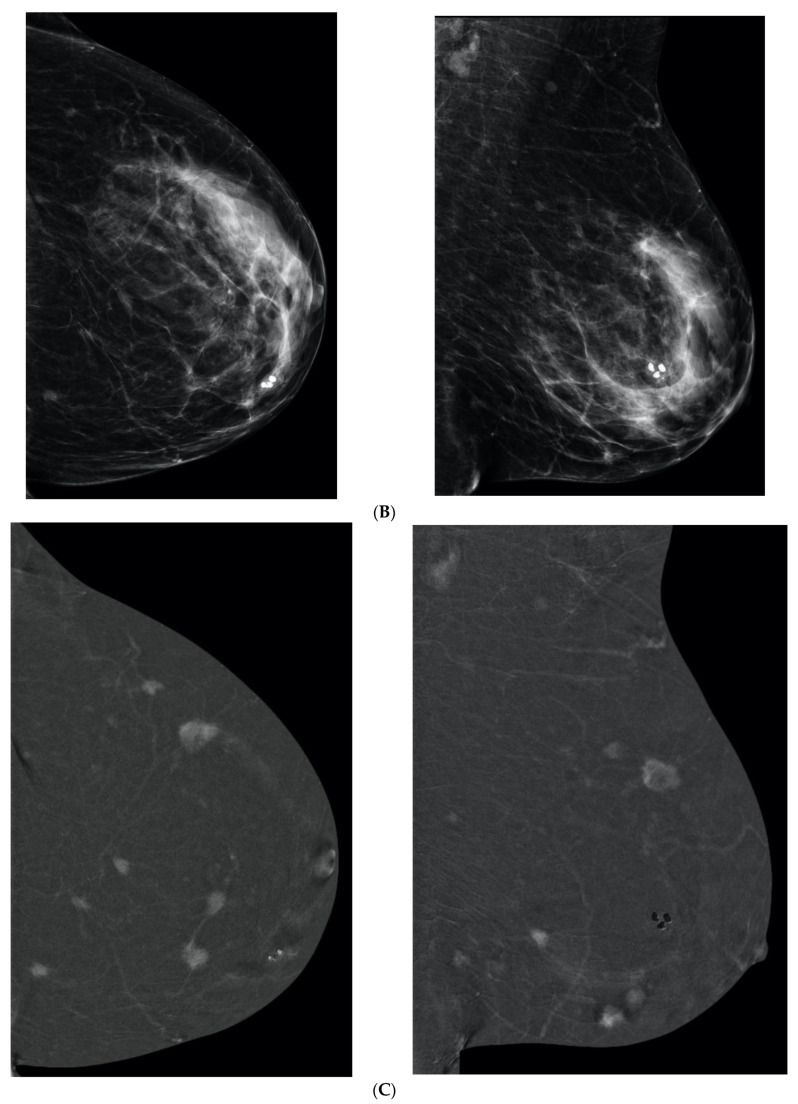
Courtesy of Dr. Anat Kornecki. This 71-year-old woman with BI-RADS C density was recalled from screening because of architectural distortion in the left upper outer quadrant. (**A**) Screening CC and MLO views, with area of distortion circled. (**B**) low-energy CC and MLO views. High-energy views not shown. (**C**) subtraction CC and MLO views, showing multiple areas of enhancement. Note that 5 lesions are in the medial breast, which was not dense mammographically. The two lesions furthest apart were core biopsied, and both confirmed malignant. Mastectomy was performed, and showed 5 foci of invasive lobular carcinoma.

The high energy image is uninterpretable (Figure 8), but is used to subtract the low-energy from, which subtracts out the normal tissue and leaves just the contrast-containing vessels and vascular tissues visible, including cancers [123]. As with MRI, this is an example of “functional imaging”, with demonstration of abnormal “leaky” vessels suggestive of malignancy. 

Both the low-energy and subtraction (also known as recombined or iodine) images are required for interpretation. Areas of suspicious calcification on the low-energy images may not have corresponding abnormal vascularity on the subtraction, and conversely, a suspicious finding on the subtraction may not have a correlate on the low-energy image, especially if it is due to a lesion obscured by dense tissue. Amir has shown that a low-energy finding with associated enhancement on the subtraction image, or a finding with a correlate on US or MRI is, predictive of malignancy. Calcifications with associated enhancement had a high malignancy rate. However, 50% of the lesions that enhanced on the subtraction images with no correlate on the low-energy were malignant [124]. Therefore, negative CEM is not sufficient to avoid performing biopsy of suspicious calcifications. Figure 8 shows an example of much more cancer seen on a CEM examination, than was visible on the screening mammogram.

CEM has similar sensitivity to MRI at considerably lower cost. Cheung showed that by using CEM in addition to mammography, sensitivity increased from 71.5% to 92.7% and specificity from 51.8% to 67.9% [125]. Fallenberg reported that 13 of 14 cancers (93%) that were occult on mammography but seen on CEM were in women with dense breasts [126]. 

The radiation is higher than that of digital mammography or DBT, but is within the allowable range [127,128,129]. Implementing CEM in a practice requires time for consenting, preparing the injector and starting the IV. IV setup is the same as for MRI, but the CEM examination and interpretation are faster than MRI. Contrast reactions can occur. Most are mild and resolve spontaneously. More moderate reactions occur in less than 1% and personnel must be appropriately trained to manage these. Fatal outcomes are extremely rare with low-osmolality contrast agents [130,131]. 

CEM has shown potential for screening women eligible for MRI who have a contraindication to it, or choose to avoid or cannot access MRI [132,133,134]. In addition to the ACR recommendations for screening high-risk women, the National Comprehensive Cancer Network (NCCN) now recommends consideration of contrast-enhanced mammography or whole breast ultrasound for those who qualify for, but cannot undergo MRI [135]. CEM outperforms mammography and US at screening, so women who have CEM do not need supplemental US [135,136].

CEM may be better than MRI at depicting lesions that enhance late because of the longer time after contrast is given before imaging in CEM, and the different structures of Gadolinium and iodinated contrast [126]. CEM-directed biopsy has received FDA and Health Canada approval, but is not yet commercially available [137,138,139]. For now, if a suspicious lesion is detected. Guidance with either US or MRI is required.

CEM has false alarms, like any other screening test, most commonly fibroadenomas, papillomas, and hyperplasia [140]. 

A multicenter trial of CEM is in the planning stages. The “Contrast Enhanced Mammography Imaging Screening Trial” (CMIST) will enroll 2500 average-to-intermediate risk women aged 40–75 years for 2 consecutive years of screening with DBT, US and CEM and one year of follow-up. Performance metrics will be compared, in addition to the tumor biologic characteristics of invasive cancers and DCIS detected at CEM and DBT plus screening US [119,141].

The ACR has released a lexicon for CEM as a supplement to the 2013 ACR BI-RADS mammography [142]. 

## 9. Molecular Breast Imaging (MBI)

MBI is another functional imaging test. It uses intravenous Tc-99 m sestamibi, which is taken up by cancers more than by normal breast tissue. The dose of intravenous contrast, initially 20 to 30 mCi (740 to 1110 MBq) administered in studies using older breast-specific gamma imaging technology, has been updated to 8 mCi (296 MBq) used with newer semiconductor-based, dedicated systems that allow direct-contact breast positioning, improved spatial resolution, and now biopsy capability [143]. Soon after the injection (within 5 min), the breast is positioned similarly to mammography between two Gamma camera detectors with light compression. Each image takes 10 min, so the total examination time exceeds 40 min. The effective dose is approximately 2 mSv for MBI versus 0.5 for DM or DBT with synthetic reconstructions; 2 mSv is considered to be within safe limits [144]. Unlike low-dose radiation from mammograms, which is limited to the breasts, the radiation from this test is to the whole body, especially the pelvis. 

Radionuclide injection is typically performed in a Nuclear Medicine department. Positioning for imaging may be performed by mammography technologists, or by nuclear medicine technologists who have been trained in mammography, and supervised for a minimum 25 cases [145]. Interpretation by radiologists specialized in breast imaging is recommended, to facilitate correlation with other imaging modalities [146].

In single-institution studies, adding MBI to 2D mammography in women with dense breasts detected an additional 6.5–8.8 invasive cancers per 1000 screened, with modest increases in recall rate (6% to 8%) at a lower cost-per-cancer detected than mammography alone, and with a PPV of 33% [146,147]. 

Density MATTERS (Molecular Breast Imaging and Tomosynthesis to Eliminate the Reservoir of Cancers) is an ongoing multicenter trial comparing DBT to MBI in women aged 40–75 years with dense breasts. It will also assess for change in the advanced cancer rate by performing two consecutive annual MBI scans. Their preliminary report showed cancer detection rates per thousand of 1.9 for DBT vs. 11.2 for MBI (ICDR of 9.3 for MBI). PPV was 8% for DBT, 26% for MBI and 21% for the combination of DBT and MBI [148].

**Figure 9:**
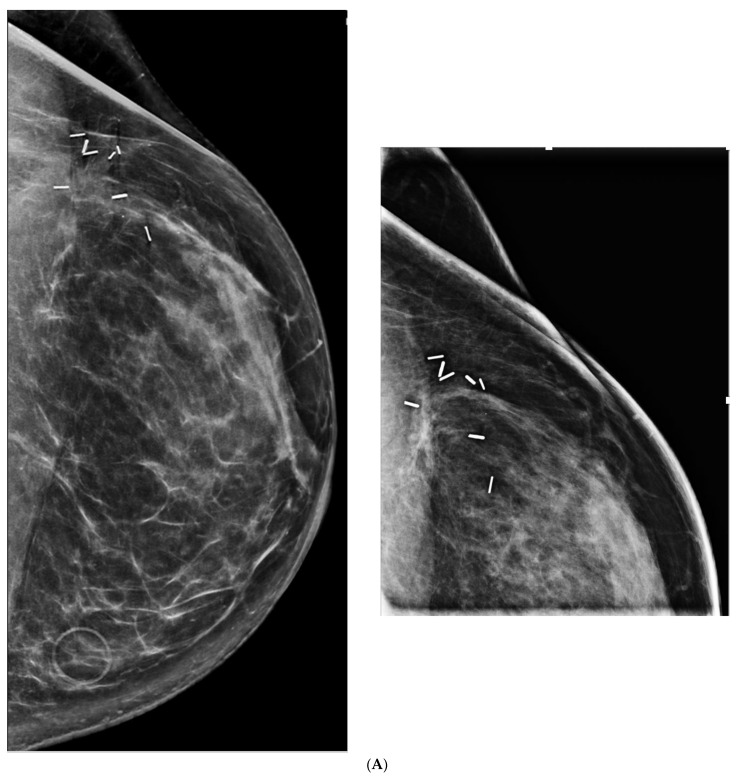

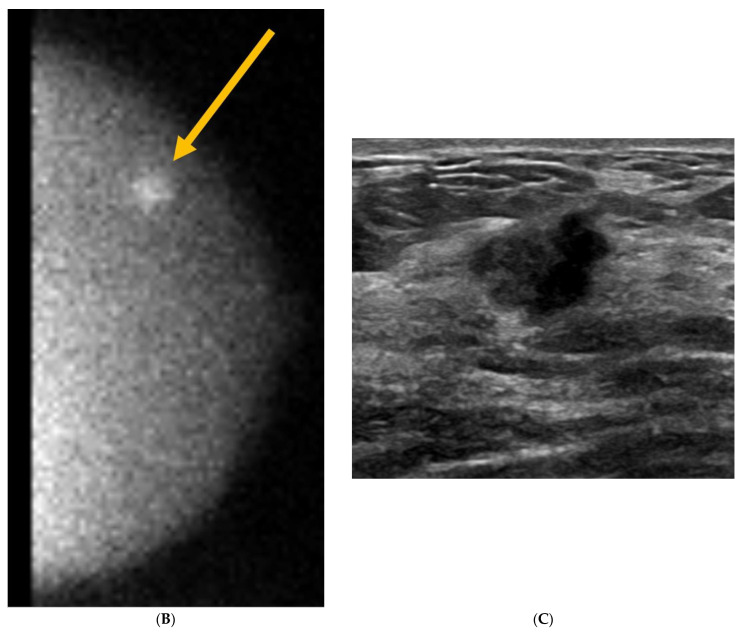
Courtesy of Drs. Deborah Rhodes and Carrie Hruska. Negative mammogram in a woman post lumpectomy. (**A**) cc views, routine and spot magnification. Tomosynthesis (not shown) was also negative. Notably, the breast is category B, not dense. (**B**) Recurrent carcinoma (arrow) detected on MBI (**C**) Targeted US demonstrates the cancer.

Hruska asserts that concern regarding the radiation dose in MBI has hindered its wider use despite compelling evidence of its benefits [149], and points out that benefit to risk ratios for MBI studied by Brown and Covington [150] and Hendrick [151] are superior to mammography in specific scenarios. Figure 9 shows a cancer seen on MBI that was not detected on FFDM or DBT. She argues that MBI “consistently shows it is doing the task that we wish it to do—finding invasive cancers occult on mammographic screening, at a relatively low false-positive rate and low cost”. It is recommended that when used in conjunction with MBI, DBT be performed with synthetic 2D rather than full field digital mammography (FFDM 2D) to reduce dose.

Performance of MBI requires cooperation between nuclear medicine and breast-imaging departments. In the USA, nuclear medicine is part of the radiology residency [152], but this is not the case in Canada. Some radiologists do additional training in Nuclear Medicine, but rarely interpret breast-imaging examinations, so MBI is not currently practiced anywhere in Canada.

## 10. Artificial Intelligence (AI)

**Figure 10:**
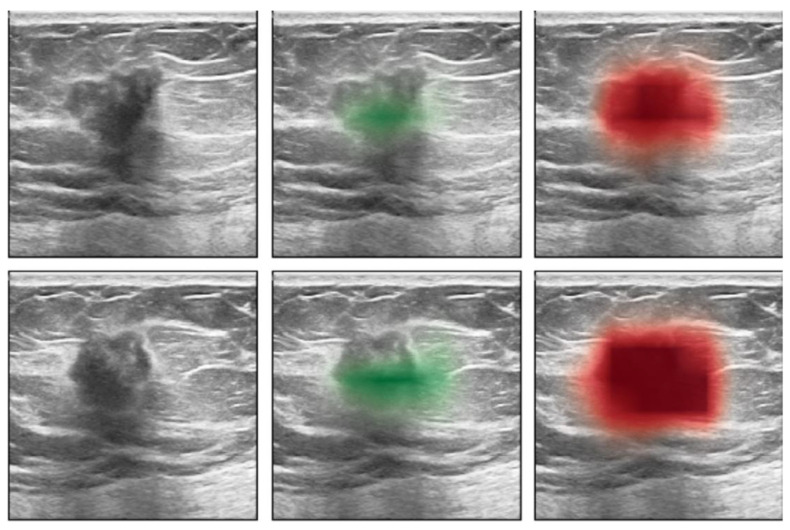
From Shen et al. [156] Case B from their Figure 3. No changes have been made. https://creativecommons.org/licenses/by/4.0/legalcode (accessed 12 March 2022). Sagittal (**top row**) and transverse views (**lower row**) of a biopsy proven-cancer. The images on the (**left)** are the B-mode images. The saliency maps indicate the predicted locations of benign (**middle**) and malignant (**right**) findings.

AI shows promise to improve screening in all women, including those with dense breasts. Romero-Martin et al. showed that stand-alone use of AI for digital mammography screening had non-inferior sensitivity and a lower recall rate for 2D mammography than by single or double-reading by dedicated breast radiologists (but not for DBT) [153]. Shoshan et al. tested an AI system for DBT, and showed that it could reduce radiologists’ workload by almost 40%, by filtering out negative DBT screening studies with non-inferior sensitivity, and 25% reduction in recalls [154]. However, in the accompanying editorial, Philpotts rightly points out that although “non-inferior”, of the 459 detected cancers, 4 detected by the radiologists were missed by the AI. She suggests that false negative AI would not be acceptable to women [155]. Shen et al. described an AI system for breast ultrasound that maintains its sensitivity, while reducing the recall rate by 37% and reduces biopsy recommendations by 28% [156] (Figure 10). As Fuchsjäger points out, “assistance from AI could yield a considerable workload reduction if it could be shown to reliably identify negative mammograms and make US unnecessary”. However, prior to AI being implemented without radiologist input, legal issues will have to be addressed, both for medical decision-making and data security [157]. 

Mango et al. described a system of AI-based decision support for breast lesion assessment, and showed improved accuracy while reducing inter- and intra-observer variability [158]. 

All of the modalities discussed are potentially useful in finding cancers missed on mammograms, but it would be helpful to determine which patients are at higher risk of interval cancer. Wanders et al. searched for ICs diagnosed after a normal screening mammogram among 1,163,147 women in the Dutch screening program. There were 2222 women diagnosed with IC within 20 months of a negative mammogram. The control group was 4661 age-matched women who had at least 2 years of normal follow-up findings after negative screening findings. Using software to calculate breast density, combined with an AI cancer detection system based on deep convolutional neural networks, they found a higher proportion of women who subsequently developed IC compared with either one, alone [159]. We can expect to see much more research on AI to refine how to find breast cancer as early as possible for all women. 

## 11. Summary of Benefits vs. Risks

Supplemental screening modalities vary in the degree of associated risks, including cost, anxiety from recalls, including those that lead to negative biopsies, exposure to ionizing radiation, adverse events from injected agents, and “overdiagnosis”.

DBT is widely used in the USA, performed most often as part of the screening mammogram rather than as a supplemental screen. It increases cancer detection compared with 2D mammography, but finds more slowly growing cancers than contrast-based techniques, and about half as many additional cancers as US. However, just as important, it reduces recall rate. So its use in initial screening is justified given the equivalency of dose when obtained with synthetic 2D. 

Ultrasound is widely available, uses no ionizing radiation or injection, and cost is generally low. Its high initial recall rate increases cost and anxiety, but recalls decrease with experience and availability of priors for comparison. Sensitivity is modest compared to other supplemental modalities. However, the most recent publication showed an impressive ICDR of 7/1000 on the prevalent screen [56]. 

At the other end of the spectrum, MRI is the most sensitive and expensive, but not as widely available, especially outside the USA [160]. It uses no ionizing radiation but requires IV gadolinium, which accumulates in the bone, skin, solid organs, and brain. Although there are currently no known harmful effects, Neal points out that, “the absence of evidence does not equal the absence of risk” [98]. MRI’s longer-term low interval cancer rate may allow less frequent examinations. The cost should drop considerably with wider implementation of abbreviated MRI. Further study with DWI may decrease the need for contrast.

CEM has sensitivity approaching that of MRI, uses standard mammography equipment and is becoming more widely-available. It requires injection, but uses iodinated contrast; not gadolinium.

Molecular breast imaging (MBI) uses injected radioactive material and has an effective dose approximately four times that of digital mammography or DBT. Contrast reactions are rare, but it requires longer exam time and is not widely available.

The choice of supplemental screening should be based on an individual woman’s risk profile. In 2010, Kuhl et al. used data from the EVA trial to compare the cancer detection rates of clinical breast examination, mammography, ultrasound, and MRI, either alone or in various combinations, for screening women at ≥20% lifetime risk for breast cancer, irrespective of breast density. Cancer yields were: mammography: 5.4/1000, US: 6/1000, mammography plus US: 7.7/1000, MRI alone: 14.9/1000, MRI plus US: 14.9/1000, and MRI plus mammography 16/1000 (not statistically significantly increased) [92].

More recently, Berg has created a chart summarizing the ICDR in various breast densities, the additional recalls, and the impact on the IC rate of DBT, US, MBI, CEM, MRI and AB MRI, best viewed online at https://densebreast-info.org/screening-technologies/cancer-detection-by-screening-method/ [161].

Panels in North America that create breast cancer screening guidelines [162,163] have traditionally relied exclusively on decades-old RCTs, which did not include considerations of menopausal status, breast density, race/ethnicity, current hormone therapy, and other risk factors. Because they ignore well-performed observational studies and surrogate endpoints, they state that there is insufficient evidence for supplemental screening for breast cancer for women with dense breasts using other imaging modalities.

Guidelines for breast cancer screening in the USA vary considerably, depending on the issuing organization. There is still a moratorium on the United States Preventive Services Task Force guidelines that recommend starting mammography screening at age 50. Most screening in the USA is ad hoc, whereas elsewhere it is largely performed in organized programs. In most Canadian jurisdictions and many European countries, mammography screening is biennial beginning at 50 for women at average risk. Women who are premenopausal after age 50, post-menopausal women on combined hormone therapy, obese women, and women with dense breasts are at increased risk of getting breast cancer, and increased risk of interval cancer [5,6,7,8,9,10,11,12,13,14].

Efforts are underway to refine which women are at low, medium or high risk, and whether there is a subset of women who are at such low risk that they can be screened less often. AI may prove effective at discriminating which women are at high risk of IC. Until these studies are completed and to provide equity, organized screening programs that screen less often than annually should consider decreasing the screening interval for mammography for women at higher-than-average risk, and consider issuing recommendations supplemental screening for women at increased risk, and for women with dense breasts.

## Abbreviations

RCT	Randomized Controlled Trial
ACR	American College of Radiology
BI-RADS	Breast Imaging Reporting and Data System
FFDM	Full Field Digital Mammography
IC	Interval Cancer
ER	Estrogen Receptor
PR	Progesterone Receptor
US	Ultrasound
DBT	Digital Breast Tomosynthesis
MRI	Magnetic Resonance Imaging
CEM	Contrast-Enhanced Mammography
MBI	Molecular Breast Imaging
DCIS	Ductal Carcinoma In-situ
ICDR	Incremental Cancer Detection Rate
ACRIN	American College of Radiology Imaging Network
PPV	Positive Predictive Value
J-START	Japan Strategic Anti-cancer Randomized Trial
FDA	United States Food and Drug Administration
2D	Two Dimensional
MLO	Mediolateral Oblique
CC	Craniocaudal
QUALYs	Quality-Adjusted Life-Years
EUSOBI	European Society of Breast Imaging
DW MRI	Diffusion-Weighted MRI
CE MRI	Contrast-enhanced MRI
AB MRI	Abbreviated MRI
CT	Computed Tomography
IV	Intravenous
kVp	Kilovoltage peak
